# One-Pot Synthesis and High Electrochemical Performance of CuS/Cu_1.8_S Nanocomposites as Anodes for Lithium-Ion Batteries

**DOI:** 10.3390/ma13173797

**Published:** 2020-08-28

**Authors:** Lin-Hui Wang, Yan-Kun Dai, Yu-Feng Qin, Jun Chen, En-Long Zhou, Qiang Li, Kai Wang

**Affiliations:** 1 College of Information Science and Engineering, Shandong Agricultural University, Taian 271018, China; linhuiwang@sdau.edu.cn (L.-H.W.); chenj@sdau.edu.cn (J.C.); 2 College of Chemistry and Material Science, Shandong Agricultural University, Taian 271018, China; dyk20000829@163.com (Y.-K.D.); chemelzhou@sdau.edu.cn (E.-L.Z.); 3 College of Physics, University-Industry Joint Center for Ocean Observation and Broadband Communication, Qingdao University, Qingdao 266071, China; 4 College of Electrical Engineering, Qingdao University, Qingdao 266071, China; wangkai@qdu.edu.cn

**Keywords:** Cu_x_S nanoparticles, lithium-ion batteries, one-pot synthesis, high electrochemical performance, anodes

## Abstract

CuS and Cu_1.8_S have been investigated respectively as anodes of lithium-ion batteries because of their abundant resources, no environment pollution, good electrical conductivity, and a stable discharge voltage plateau. In this work, CuS/Cu_1.8_S nanocomposites were firstly prepared simultaneously by the one-pot synthesis method at a relatively higher reaction temperature 200 °C. The CuS/Cu_1.8_S nanocomposites anodes exhibited a high initial discharge capacity, an excellent reversible rate capability, and remarkable cycle stability at a high current density, which could be due to the nano-size of the CuS/Cu_1.8_S nanocomposites and the assistance of Cu_1.8_S. The high electrochemical performance of the CuS/Cu_1.8_S nanocomposites indicated that the Cu_x_S nanomaterials will be a potential lithium-ion battery anode.

## 1. Introduction

Because of the rapid development of human society, energy demand is increasing. Fossil fuels have gradually dried up and caused severe environmental pollution. Clean and efficient energy devices play a vital role in coping with global warming and energy crisis, such as lithium-ion batteries [1,2,3,4,5,6,7,8,9,10,11,12], supercapacitors [13,14,15,16], nanogenerators [17,18,19]. Rechargeable lithium-ion batteries have received lots of attention because of the rapid development of rechargeable electrical vehicles and portable electric devices [20,21,22,23]. The rechargeable lithium-ion batteries with excellent performance should have a high energy density, long discharge-charge cycle life, perfect rate capability, and no environmental pollution. Traditional graphitic carbon materials are widely used as anodes because of its good electrochemical stability, and abundant reserves. However, the relatively low theoretical capacity of only 372 mAh g^−1^ is impeding the further applications [24,25,26,27]. Transition-metal chalcogenides are a promising choice for developing new anodes of lithium-ion batteries for their much higher theoretical capacity [20,21,22,25,26,27,28]. A series of transition-metal oxides and transition-metal sulfides have been investigated and exhibited many exciting properties [20,21,22,23,24,25,26,27]. Among these anode materials, transition-metal sulfides have attracted more and more attention because of the excellent electronic conductivity, high specific capacity, and environment friendly [20,21,22,23,24,25]. Copper and cuprous sulfides have received extensive attention and research because of their abundant resources, no environment pollution, a flat and long discharge voltage plateau, high theoretical capacity, and high electrical conductivity (10^−3^ S cm^−1^) [21,22,23,24,25,29,30,31,32,33,34,35,36].

In order to solve the volume expansion and to shorten the diffusion length of lithium ions, various types of Cu_x_S nano/micro-morphologies have been prepared by many kinds of methods [21,22,23,24,25,26,27,29,30,31,32,33,34,35,36,37,38,39,40,41], such as nanoparticles [31,38], nanorods [21], hollow spheres [22], mircroflowers [24], nanotubes [29], nanosheets [30,33,37], hierarchical [34], and other nanocomposites [24,25,32,33,34,35,36]. Zhao et al. reported that at a high current rate, the specific capacity of CuS electrode was more than 370 mAh g^−1^ [21]. Du and Li et al. prepared small-sized CuS nanoparticles/N, S co-doped rGO composites, and the obtained CuS@N/S-G-C6 electrode delivered a reversible capacity up to 530 mAh g^−1^ at 2 A g^−1^ after 1000 cycles, and 603.5 mAh g^−1^ at 200 mA g^−1^ after 300 cycles [31]. Jiao and Xu et al. prepared hollow CuS nanoboxes which achieved outstanding rate performance and an ultra-long discharge-charge cycle life [42].

Recently, Cu_1.8_S has also been paid significant attention in sodium-ion batteries and lithium-ion batteries owing to its crystallographic stability and narrow band gap of 1.2 eV (better electrical conductivity) [35,43,44]. Zaworotko and Ryan et al. reported that Cu_1.8_S/C-500 nanowire composites exhibited the best performance, and showed a specific capacity up to 220 mAh g^−1^ after 200 cycles [43]. Kang and Lee et al. reported that a nanoporous Cu_1.8_S-C/C core/shell anode structure with a high surface area achieved a specific capacity of 372 mAh g^−1^ after 110 cycles with a performance of ~93% capacity retention [35]. Chen and Lou et al. reported that a designed 3D core-shell Cu_1.8_S/C@MoS_2_ nanocomposite exhibited good stability, outstanding rate performance, and high reversible capacity, because of the assistance of Cu_1.8_S/C [37].

Graphene and carbon nanotubes were added to CuS to improve the electrochemical properties in many articles [24,25,31,32,33,37,38,39,40,41]. However, the synthesis methods are complicated and Graphene and carbon nanotubes are expensive. So it is necessary to introduce more suitable materials to improve the electrochemical properties of CuS. The synthesis methods and the electrochemical properties of CuS/Cu_2_S nanocomposites [21] and Cu_2_S/Cu_1.8_S nanocomposites [22,45,46] have been investigated. However, the nanocomposites of CuS/Cu_1.8_S have not been prepared and studied. It was reported that CuS could be reduced to Cu_1.8_S at a high temperature and a high pressure [45]. In this work, we firstly fabricated CuS/Cu_1.8_S nanocomposites by a simple one-pot synthesis method at a correspondingly higher reaction temperature 200 °C. The CuS/Cu_1.8_S nanocomposites anodes exhibited high initial discharge capacity (1555 mAh g^−1^ at 100 mA g^−1^ and 1130 mAh g^−1^ at 267 mA g^−1^), excellent cycle stability at high current density (average 425 mAh g^−1^ at 267 mA g^−1^ during 1000 cycles, 472 mAh g^−1^ after 700 cycles, and 325 mAh g^−1^ after 1000 cycles), and excellent rate performance, which indicated that the Cu_x_S nanomaterials will be a potential lithium-ion battery anode.

## 2. Materials and Methods

### 2.1. Preparation of CuS/Cu_1.8_S Nanocomposites

**Figure 1:**
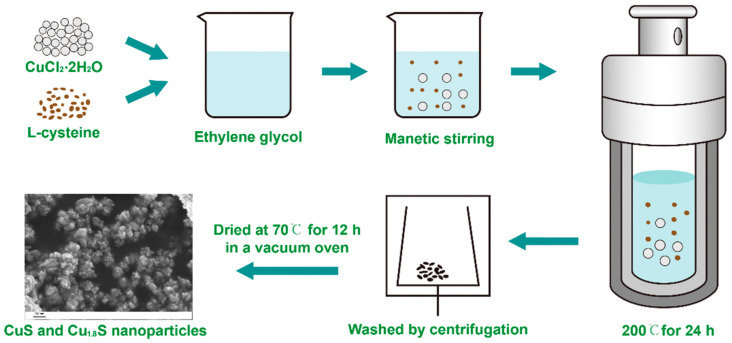
The schematic illustration of the preparation of CuS/Cu_1.8_S nanocomposites.

The schematic illustration of the preparation of CuS/Cu_1.8_S nanocomposites is shown in Figure 1. Thereby, 489.7 mg L-cysteine and 681.92 mg CuCl_2_·2H_2_O were sequentially added into 70 mL ethylene glycol. After1 h of magnetic stirring, the dissolved solution was put into two Teflon-sealed autoclaves (50 mL), which were heated to 200 °C and maintained for 24 h. When naturally cooled down to room temperature, the black precipitates were rinsed by ethanol and deionized water, in turn, many times by centrifugation (10000 rpm 10 min) until the solution is clear. Finally, the obtained samples were dried in a vacuum oven at 70 °C for 12 h.

### 2.2. Structure and Morphology of CuS/Cu_1.8_S Nanocomposites

Scanning electron microscope (SEM, GeminiSEM300, Zeiss, Oberkochen, Germany) and X-ray diffraction (XRD, SmartLab, Rigku, Tokyo, Japan) were used to characterize the structure, morphology, and composition of the samples. The XRD measurements were performed in the rage of 20° to 80° at a measuring rate of 3°/min using a Cu Kα radiation.

### 2.3. Lithium-Ion Battery Performance of CuS/Cu_1.8_S Nanocomposites

The working anodes were mixed with as-prepared black powders, carbon black, and carboxymethyl cellulose (CMC) dissolved in deionized water with a weight ratio of 7:2:1. The obtained slurry was evenly coated on a copper foil and dried in a vacuum oven at 70 °C for 12 h. And then, the obtained coated foil was punched into disks with a diameter of 12 mm. The weight of each disk sheet was measured to calculate the mass of active materials. The mass density of the active material was calculated at about 0.4 mg cm^−2^.

The electrochemical measurements were carried out on CR-2032 coin-type cells. The half-cells were assembled in an argon glove box. The concentrations of oxygen and moisture in the argon glove were lower than 1 ppm. The Celgard 2250 film and lithium metal disk were selected as diaphragm and counter electrode, respectively. The electrolyte was an organic electrolyte of 1 M LiPF6, which is dissolved in a mixture of dimethyl ethyl carbonate (DEC) and ethyl carbonate (EC) with a volume ratio of 1:1.

Land-ct2001A battery measuring system was used to test the charge-discharge characteristics under different current densities with a potential range of 0.01 V–3.0 V. CHI660E electrochemical workstation was used to get the cyclic voltammetry (CV) measurements with a scanning rate of 0.1 mV s^−1^ and in a potential range between 0.01 V and 3.0 V. The electrochemical impedance spectroscopy (EIS) was also obtained by the CHI660E electrochemical workstation with a frequency range of 10^−2^ Hz–10^5^ Hz. All the cells measured were set more than 24 h to ensure total penetration of the electrolyte into the diaphragm, and all measurement results were obtained at room temperature.

## 3. Results and Discussion

### 3.1. Morphology and Structure of CuS/Cu_1.8_S Nanocomposites

**Figure 2:**
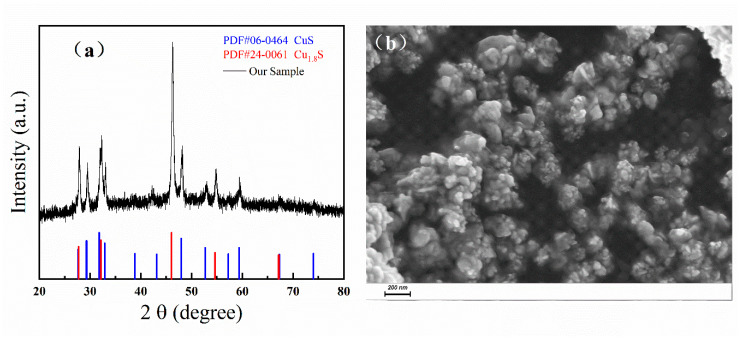
(**a**) XRD patterns of the sample and the stand PDF cards of CuS and Cu_1.8_S. (**b**) The SEM image of our sample with a 200 nm scale bar below.

Figure 2a shows the XRD patterns of our samples, which is in good agreement with the standard cards of PDF No. 06-0464 (CuS) and No. 24-0061 (Cu_1.8_S), demonstrating that the as-prepared samples were composed of CuS and Cu_1.8_S. No other characteristic peaks for impurities were observed. The sharp diffraction peaks in the patterns indicated the good crystallinity of our samples. Different peaks at 27.8°, 29.4°, 31.9°, 33.0°, 48.1°, 52.9°, and 59.4° corresponded to the (101), (102), (103), (006), (110), (108), and (116) crystal plane of CuS [23,24,25,39,40,41,42,43,44,45,46]. Furthermore, peaks at 27.8°, 32.3°, 46.2°, and 54.8° corresponded to (111), (200), (220), and (311) crystal plane of Cu_1.8_S [23,44,45]. The peak of the (101) crystal plane of CuS and the (111) crystal plane of Cu_1.8_S could overlap to 27.8°. The average sizes of crystal particles can be obtained by the Scherrer Equation [25,47]:

(1) $$D_{(\mathrm{hkl})}=\frac{k\lambda }{b_{(\mathrm{hkl})}\cos\theta }$$

where *λ* is the wavelength of the X-ray applied in the measurement, *k* is a constant fact 0.9, *θ* is the diffraction angle, and *b* is the full width at half maximum (FWHM). The sizes of CuS/Cu_1.8_S particles were estimated to be 23 nm (*D*_(110)_) and 22 nm (*D*_(220)_), which are nearly equal to each other.

The morphology of as-prepared samples was further investigated using SEM, as is shown in Figure 2b. It can be seen from the SEM image that the as-prepared samples consisted of nanoparticles with sizes in the range of 10 nm–100 nm, which was consistent with the results calculated by the Scherrer equation. The mole ratio of CuS and Cu_1.8_S was measured by Energy Dispersive Spectrometer (EDS). The EDS results are shown in Figure S1 and Table S1 of supplementary data, and the mole percentage of CuS is about 88%.

### 3.2. Electrochemical Performance of CuS/Cu_1.8_S Nanocomposites

**Figure 3:**
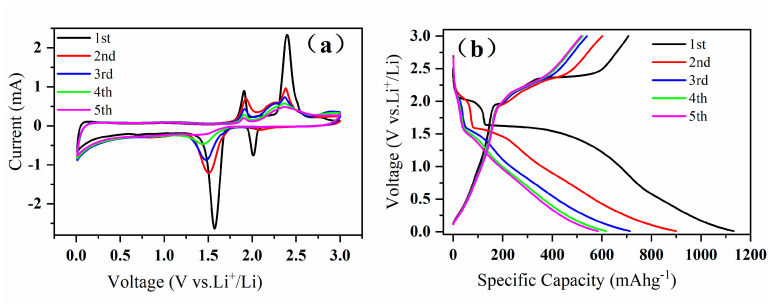
(**a**) The initial five cyclic voltammetry curves with a scanning rate of 0.1 mV s^−1^ and in a potential range between 0.01 V and 3.0 V. (**b**) The initial five discharge and charge curves at a current density of 267 mA g^−1^.

In order to further understand the electrochemical process of CuS/Cu_1.8_S nanocomposites, the cyclic voltammetry (CV) curves were measured with a scanning rate of 0.1 mV s^−1^ and in a potential range between 0.01 V and 3.0 V. As shown in Figure 3a, during the first cathodic sweep (lithiation), two prominent reduction peaks at 2.0 V and 1.6 V were observed, which could be attributed to the process of CuS to Li_x_CuS and the conversion of Li_x_CuS to Cu and Li_2_S, respectively. During the first anodic sweep (delithiation), two obvious oxidation peaks at 1.9 V and 2.4 V were observed, which denoted the reversible process related to the cathodic reactions [24,25,27,40,41,47]. The reduction peak at 2.0 V transferred to 2.1 V in the second scan and faded in the subsequent scans, which has also been observed in other reports [25,40,48]. Furthermore, the reduction peak at 1.6 V transferred to lower potential values gradually, which could indicate the increase of energy and the polarization of the electrodes [48]. Figure 3b shows the corresponding initial five galvanostatic discharge-charge curves of CuS/Cu_1.8_S nanocomposites at 267 mA g^−1^. In the first discharge process, two potential plateaus appeared at about 2.1 V and 1.6 V, and in the first charge process, two potential plateaus appeared at around 1.9 V and 2.3 V. These are matched with the redox peaks (redox reactions) of the first CV curve very well [40,41,47,48]. Compared with the CV curves, the discharge potential plateau at 2.1 V also gradually disappeared in the subsequent cycles. Importantly, we obtained the relatively higher initial discharge capability of 1130 mAh g^−1^ and charge capability of 707 mAh g^−1^ [22,23,24,25,40,41,47,48], which could be ascribed to the formation of solid electrolyte interface (SEI) and the existence of Cu_1.8_S [31,32,33,34,35,36,37]. The discharge capacities of the fourth and fifth cycles were about 610 mAh g^−1^ and 580 mAh g^−1^, which are also higher than the theoretical capability 560 mAh g^−1^ of CuS. This extra capacity has been widely observed transition metal compounds [49], which can be attributed to the formation/decomposition of polymeric gel-like films around the transition metal particles [50], the interface lithium storage [51,52], and the surface conversion of LiOH to Li2O and LiH [53]. In the following cycles, the curves tended to overlap, which showed the outstanding cyclic stability of the CuS/Cu_1.8_S nanocomposites.

**Figure 4:**
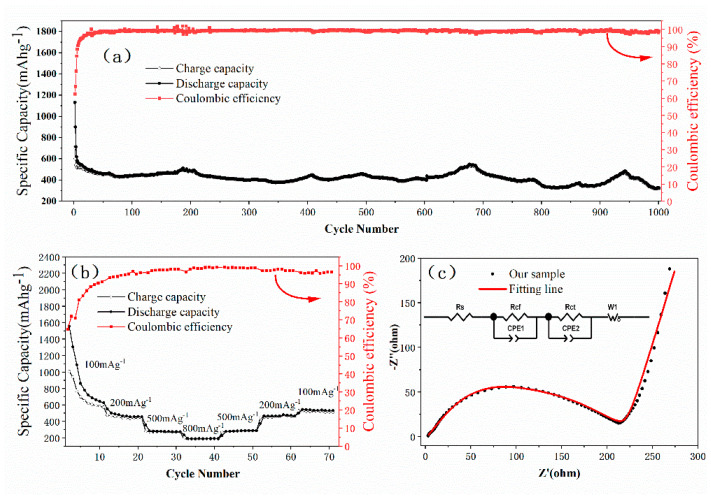
(**a**) The cycling performance and the corresponding coulombic efficiency at a current density of 267 mA g^−1^. (**b**) The rate capability and the corresponding coulombic efficiency at different current densities. (**c**) The electrochemical impedance spectroscopy with a frequency range of 10^−2^ Hz–10^5^ Hz.

Figure 4a depicts the cycling performance of the CuS/Cu_1.8_S nanocomposites at 267 mA g^−1^. As shown in Figure 4a, the initial coulombic efficiency (CE) was 62.5% and increased to around 99% rapidly. At the same time, the specific capacity decreased from 1130 mAh g^−1^ to about 450 mAh g^−1^. The values of specific capacity and the corresponding coulombic efficiency almost maintained to 1000 cycles with a small range of fluctuation, which demonstrated excellent cycling stability and relatively high lithium storage capacity. A fluctuation of the capacity can be seen in Figure 4a. It is found that the specific capacity of some copper sulfide materials gradually increases with the charge-discharge process due to a possible activation process in the electrode materials [27,30,32,33,41,47]. In the experiments, the particle size ranges from 10–100 nm. The inside of the larger particles may not contribute to the capacity. With the charge-discharge process, the larger particles may decompose into smaller particles, which will provide more specific surface area and more effective active materials. In addition to the changes of the measuring temperature [25], the increased specific surface area and effective active materials may cause the fluctuation of specific capacity.

The electrochemical performances of CuS-based electrodes as anode materials for lithium-ion batteries were listed in Table 1. Compared with the previously reported results, the CuS/Cu_1.8_S nanocomposites in our work has good lithium-storage capability, high initial capacity, and good cycling stability. The CuS/Cu_1.8_S nanocomposites electrode could maintain the reversible capacity of 440 mAh g^−1^ after 100 cycles, 450 mAh g^−1^ after 500 cycles, 470 mAh g^−1^ after 700 cycles, and about 325 mAh g^−1^ after 1000 cycles. During the 1000 cycles, the average discharge capability and charge capability were 425 mAh g^−1^ and 420 mAh g^−1^ respectively. The high electrochemical performances of CuS/Cu_1.8_S nanocomposites electrode in our work could be attributed to the nano-size of the composites and the assistance of Cu_1.8_S [22,23,24,25,26,27,37,38,42,43].

As shown in Figure 4b, the rate capability of CuS/Cu_1.8_S nanocomposites anodes was further tested at a series of current densities. Despite an obvious fading of capacity during the initial several cycles, the CuS/Cu_1.8_S nanocomposites electrodes exhibited extraordinary stability and reversible capabilities in subsequent cycles. The reversible capacities had an obvious stepwise trend with the changing of current densities. The capacities were 1555 mAh g^−1^, 485 mAh g^−1^, 275 mAh g^−1^, and 195 mAh g^−1^ at 100 mA g^−1^, 200 mA g^−1^, 500 mA g^−1^, and 800 mA g^−1^, respectively. When the current densities came back to 200 mA g^−1^ and 100 mA g^−1^, the capacities could recover to 470 mA g^−1^ and 530 mA g^−1^, respectively, which implied the outstanding stability and reversibility [25,26,27,29,30,31,41,47,48,54].

In order to obtain further understanding of the enhanced electrochemical performance of CuS/Cu_1.8_S nanocomposites electrode, electrochemical impedance spectroscopy (EIS) was measured. The Nyquist plot (black dots) measured before cycling and the relative fitting line (red line) were shown in Figure 4c. And the corresponding equivalent circuit was also showed in Figure 4c. Two depressed semicircles and a straight line were found in the Nyquist plot. The intercept in the high frequency region represents the ohmic resistance (*R*_s_) of the electrode and electrolyte [54,55,56]. The small semicircle at high frequency is related to the impedance of SEI film (*R*_c__f_) [54,57]. The semicircle at high-medium frequency is related to the charge-transfer resistance (*R*_ct_) induced by the diffusion of lithium ions between electrode and electrolyte. The slope of the straight line in low frequency corresponds to Warburg impedance (*Z*_w_) relating to the diffusion of lithium ions in CuS/Cu_1.8_S nanocomposites electrode [37,40,41,45,47,48,54,55]. The Nyquist plot could be well fitted by the equivalent circuit shown in Figure 4c. The values of *R*_s_, *R*_c__f_ and *R*_ct_ are 1.8 ohm, 34.3 ohm and 175 ohm, respectively. The EIS measured after 1000 cycles and the fitting line were shown in Figure S2. The values of *R*_s_, *R*_c__f_ and *R*_ct_ increased after 1000 discharge-charge cycles, and the comparisons of *R*_s_, *R*_c__f_ and *R*_ct_ before cycling and after 1000 cycles can be seen in Table S2. Furthermore, the diffusion coefficient of Li-ions ( $D_{Li^{+}}$ ) could be obtained by the Equations as follows [54,55]:

(2) $$D_{Li^{+}}=\frac{R^{2}T^{2}}{2A^{2}n^{4}F^{4}C^{2}\sigma^{2}}$$

(3) $$Z_{\mathrm{real}}=R_{s}+R_{\mathrm{ct}}+\sigma \omega^{-1/2}$$

where *ω* is the angular frequency in the low frequency region, *σ* is the Warburg coefficient, *C* is the concentration of lithium ions, *n* is the number of transferred electrons, *F* is the Faraday constant, *T* is the measuring temperature, *A* is the surface area of the electrode, and *R* is the gas constant [54,55]. The value of *σ* was obtained by linear fitting of *Z*_real_ versus *ω*^−1/2^ Equation (3), and then the diffusion coefficient of Li-ions ( $D_{Li^{+}}=3.89\times 10^{-12}{\;\mathrm{cm}}^{2}{\;S}^{-1}$ ) was calculated by Equation (2). The relatively low *R*_ct_ and high $D_{Li^{+}}$ further indicated the enhancement of capability and rate performance of the CuS/Cu_1.8_S nanocomposites electrode [40,41,47,48,54,55], which could be attributed to the small sizes of CuS/Cu_1.8_S nanocomposites and the assistance of Cu_1.8_S [22,23,24,25,26,27,29,30,31,36,37,38,39,42,43,44,45,46]. The nano-size of the CuS/Cu_1.8_S nanocomposites could increase the specific surface area and shorten the length of lithium ions diffusion [20,21,22,23,24,25,31,32,33,34,35,36,37,38,39,40,41,42,43,44,45,46,47,48]. The existence of Cu_1.8_S might prevent the stacking of CuS and Cu particles, which could enhance the cycling stability at high current densities [24,37,43,44].

**Figure 5:**
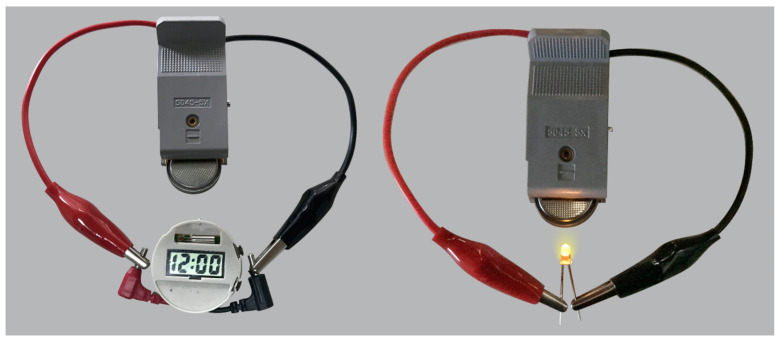
A test of a CuS/Cu_1.8_S nanocomposites half-cell after 1000 cycles illuminating an electronic watch and a LED lamp.

In addition, as shown in Figure 5, we presented a photographic image which is a test of a CuS/Cu_1.8_S nanocomposites half-cell after 1000 cycles illuminating an electronic watch and a LED lamp. The test demonstrated the high electrochemical performance and potential application of Cu_x_S nanomaterials in lithium-ion batteries.

## 4. Conclusions

In this work, CuS/Cu_1.8_S nanocomposites were firstly prepared simultaneously by the one-pot Synthesis method at a relatively high reaction temperature 200 °C. The CuS/Cu_1.8_S nanocomposites anodes exhibited a high initial discharge capacity (1555 mAh g^−1^ at 100 mA g^−1^ and 1130 mAh g^−1^ at 267 mA g^−1^), excellent cycle stability at high current density (average 425 mAh g^−1^ at 267 mA g^−1^ during 1000 cycles), and remarkable reversible rate performance, which could be attributed to the nano-size of the CuS/Cu_1.8_S nanocomposites and the assistance of Cu_1.8_S. Though the more suitable ratio of CuS to Cu_1.8_S needs to be further investigated, the high electrochemical performance of the CuS/Cu_1.8_S nanocomposites indicated that the Cu_x_S nanomaterials will be a potential lithium-ion battery anode.

## Figures and Tables

**Table 1:** Comparison of electrochemical performances of CuS and Cu_7.2_S_4_ nanocomposites with previously reported CuS-based electrodes as anode materials for lithium-ion batteries.

Electrode Materials	Initial Discharge Capacity (mAh g^−1^)	Discharge Capacity (mAh g^−1^)	Current Density (mA g^−1^)	References
CuS/Cu_1.8_S	1130	440 (100 cycles) 450 (500 cycles) 470 (700 cycles)	267	Present work
CuS	547	472 (100 cycles)	100	[21]
Cu_2_S	350	313 (100 cycles)	100	[21]
Cu_x_S	-	220 (200 cycles)	400	[22]
Cu_x_S1.2C	345	285 (200 cycles)	400	[22]
Cu_x_S1.6C	353	274 (200 cycles)	400	[22]
CuS	670	203 (100 cycles)	116	[24]
CuS@rGO	1581	1087.1 (200 cycles)	100	[25]
CuS	663	102.3 (200 cycles)	100	[25]
CuS	506	642 (270 cycles)	50	[30]
CuS@N/S-G-C6	730.3	603 (300 cycles)	200	[31]
CuS	-	162.5 (300 cycles)	200	[31]
CuS-CNT	-	477 (180 cycles)	200	[33]
CuS	-	73 (100 cycles)	200	[33]
CuS-rGO	810	451 (50 cycles)	100	[34]
CuS	559.5	94.5 (50 cycles)	100	[34]
CuS	94	93 (30 cycles)	0.2C	[36]
CuS	525	50 (10 cycles)	50	[38]
CuS/graphene	827	296 (25 cycles)	50	[38]
Cu_1.8_S/C-500	500	220 (200 cycles)	250	[43]
Cu_2-x_S	335	228 (300 cycles)	337	[45]
Cu_2-x_S@C	408	309 (300 cycles)	337	[45]
CuS-rGO	545	422 (70 cycles)	100	[40]
CuS/graphene	-	568 (100 cycles)	50	[41]
CuS	-	147 (100 cycles)	50	[41]
CuS@rGO	851	710.7 (100 cycles)	111	[47]
CuS	506	159.7 (100 cycles)	111	[47]
CuS/graphene	627	497 (100 cycles)	200	[48]
CuS	-	379 (100 cycles)	200	[48]

## Supplementary Materials

The following are available online at https://www.mdpi.com/1996-1944/13/17/3797/s1: Figure S1: The Energy Dispersive Spectrometer of CuS/Cu_1.8_S nanocomposites, Figure S2: The electrochemical impedance spectroscopy after 1000 cycles with a frequency range of 10^−2^ Hz–10^5^ Hz, Table S1: The concentration of S and Cu element of CuS/Cu_1.8_S nanocomposites, Table S2: The comparisons of *R*_s_, *R*_cf_, and *R*_ct_ before cycling and after 1000 cycles.
