# Correction: Wang et al. One-Pot Synthesis and High Electrochemical Performance of CuS/Cu_1.8_S Nanocomposites as Anodes for Lithium-Ion Batteries. *Materials* 2020, *13*, 3797

**DOI:** 10.3390/ma19040785

**Published:** 2026-02-18

**Authors:** Lin-Hui Wang, Yan-Kun Dai, Yu-Feng Qin, Jun Chen, En-Long Zhou, Qiang Li, Kai Wang

**Affiliations:** 1College of Information Science and Engineering, Shandong Agricultural University, Taian 271018, China; linhuiwang@sdau.edu.cn (L.-H.W.); chenj@sdau.edu.cn (J.C.); 2College of Chemistry and Material Science, Shandong Agricultural University, Taian 271018, China; dyk20000829@163.com (Y.-K.D.); chemelzhou@sdau.edu.cn (E.-L.Z.); 3College of Physics, University-Industry Joint Center for Ocean Observation and Broadband Communication, Qingdao University, Qingdao 266071, China; 4College of Electrical Engineering, Qingdao University, Qingdao 266071, China; wangkai@qdu.edu.cn

## References

In the original publication [1], references 1–7, 12, 13, and 35 were found to be out of the scope of this article. References 1–7 have been deleted. References 12, 13, and 35 have been updated, and their order has been adjusted to 5, 6, and 28, as shown below:5.Hong, S.-H.; Song, M.Y. Syntheses of nano-sized Co-based powders by carbothermal reduction for anode materials of lithium ion batteries. *Ceram. Int.*
**2018**, *44*, 4225–4229. https://doi.org/10.1016/j.ceramint.2017.12.002.6.Wang, D.; Yan, B.; Guo, Y.; Chen, L.; Yu, F.; Wang, G. N-doped Carbon Coated CoO Nanowire Arrays Derived from Zeolitic Imidazolate Framework-67 as Binder-free Anodes for High-performance Lithium Storage. *Sci. Rep.*
**2019**, *9*, 5934–5942. https://doi.org/10.1038/s41598-019-42371-y.28.Zhou, Y.; Tian, R.; Duan, H.; Wang, K.; Guo, Y.; Li, H.; Liu, H. CoSe/Co nanoparticles wrapped by in situ grown N-doped graphitic carbon nanosheets as anode material for advanced lithium ion batteries. *J. Power Sources* **2018**, *399*, 223–230.

With this correction, the order of some references has been adjusted accordingly.

## Error in Table

In the original publication [1], there was a mistake in Table 1 as published. The corrected Table 1 appears below:

The authors state that the scientific conclusions are unaffected. All the corrections were approved by the Academic Editor. The original publication has also been updated.

## Figures and Tables

**Table 1 materials-19-00785-t001:** Comparison of electrochemical performances of CuS and Cu_7.2_S_4_ nanocomposites with previously reported CuS-based electrodes as anode materials for lithium-ion batteries.

Electrode Materials	Initial Discharge Capacity (mAh g^−1^)	Discharge Capacity (mAh g^−1^)	Current Density (mA g^−1^)	References
CuS/Cu_1.8_S	1130	440 (100 cycles) 450 (500 cycles) 470 (700 cycles)	267	Present work
CuS	547	472 (100 cycles)	100	[21]
Cu_2_S	350	313 (100 cycles)	100	[21]
Cu_x_S	-	220 (200 cycles)	400	[22]
Cu_x_S1.2C	345	285 (200 cycles)	400	[22]
Cu_x_S1.6C	353	274 (200 cycles)	400	[22]
CuS	670	203 (100 cycles)	116	[24]
CuS@rGO	1581	1087.1 (200 cycles)	100	[25]
CuS	663	102.3 (200 cycles)	100	[25]
CuS	506	642 (270 cycles)	50	[30]
CuS@N/S-G-C6	730.3	603 (300 cycles)	200	[31]
CuS	-	162.5 (300 cycles)	200	[31]
CuS-CNT	-	477 (180 cycles)	200	[33]
CuS	-	73 (100 cycles)	200	[33]
CuS-rGO	810	451 (50 cycles)	100	[34]
CuS	559.5	94.5 (50 cycles)	100	[34]
CuS	94	93 (30 cycles)	0.2C	[36]
CuS	525	50 (10 cycles)	50	[38]
CuS/graphene	827	296 (25 cycles)	50	[38]
Cu_1.8_S/C-500	500	220 (200 cycles)	250	[43]
Cu_2-x_S	335	228 (300 cycles)	337	[45]
Cu_2-x_S@C	408	309 (300 cycles)	337	[45]
CuS-rGO	545	422 (70 cycles)	100	[40]
CuS/graphene	-	568 (100 cycles)	50	[41]
CuS	-	147 (100 cycles)	50	[41]
CuS@rGO	851	710.7 (100 cycles)	111	[47]
CuS	506	159.7 (100 cycles)	111	[47]
CuS/graphene	627	497 (100 cycles)	200	[48]
CuS	-	379 (100 cycles)	200	[48]
