# Supplementary material for: One-Pot Synthesis and High Electrochemical Performance of CuS/Cu1.8S Nanocomposites as Anodes for Lithium-Ion Batteries
Source: Materials (Basel). 2020 Aug 28;13(17):3797. doi: 10.3390/ma13173797 (PMC7503719; doi:10.3390/ma13173797)
Supplement: Supplementary file 1 [file materials-13-03797-s001.docx]

Supplementary materials

One-Pot Synthesis and High Electrochemical Performance of CuS/Cu_1.8_S Nanocomposites as Anodes for Lithium-Ion Batteries

Lin-Hui Wang ^1^, Yan-Kun Dai ^2^, Yu-Feng Qin ^1,^ *, Jun Chen ^1^, En-Long Zhou ^2^, Qiang Li ^3,^ * and Kai Wang ^4^

^1^ College of Information Science and Engineering, Shandong Agricultural University, Taian 271018, Shandong, China; linhuiwang@sdau.edu.cn (L.-H.W.); chenj@sdau.edu.cn (J.C.)

^2^ College of Chemistry and Material Science, Shandong Agricultural University, Taian 271018, Shandong, China; dyk20000829@163.com (Y.-K.D.); chemelzhou@sdau.edu.cn (E.-L.Z.)

^3^ College of Physics, University-Industry Joint Center for Ocean Observation and Broadband Communication, Qingdao University, Qingdao 266071, Shandong, China

^4^ College of Electrical Engineering, Qingdao University, Qingdao 266071, Shandong, China; wangkai@qdu.edu.cn

* Correspondence: qinyufeng@sdau.edu.cn (Y.-F.Q.); liqiang@qdu.edu.cn (Q.L.)


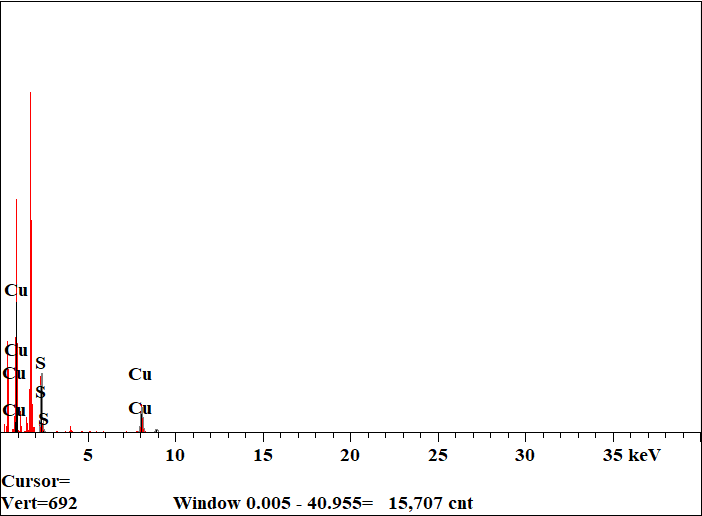


**Figure S1.** The Energy Dispersive Spectrometer of CuS/Cu_1.8_S nanocomposites.

**Table S1.** The concentration of S and Cu element of CuS/Cu_1.8_S nanocomposites.

| **Element** | **Line** | **Intensity**  **(c/s)** | | | **Concentration** | **Units** | **Error**  **2-sig** | **MDL**  **3-sig** |
| --- | --- | --- | --- | --- | --- | --- | --- | --- |
| S | Ka | | 12.79 | 31.291 | | wt.% | 1.922 | 1.216 |
| Cu | Ka | | 7.42 | 68.709 | | wt.% | 5.046 | 7.569 |
| Total | - | | - | 100.000 | | wt.% | - | - |

**

Figure S2.** The electrochemical impedance spectroscopy after 1000 cycles with a frequency range of 10^−2^ Hz–10^5^ Hz.

**Table S2.** The comparisons of *R*_s_, *R*_cf_, and *R*_ct_ before cycling and after 1000 cycles.

| **Fitted Values** | ***R*_s_ (ohm)** | ***R*_cf_ (ohm)** | ***R*_ct_ (ohm)** |
| --- | --- | --- | --- |
| Before cycling | 1.8 | 34.3 | 175 |
| After 1000 cycles | 97.05 | 1192 | 401.7 |

| 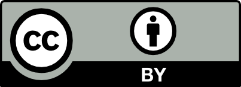 | © 2020 by the authors. Submitted for possible open access publication under the terms and conditions of the Creative Commons Attribution (CC BY) license (http://creativecommons.org/licenses/by/4.0/). |
| --- | --- |
